# The Mobile Affair

**Published:** 1898-09

**Authors:** 


### THE MOBILE AFFAIR.

THE last great cry of outrage previous to the going to press of this month's issue of the JOURNAL was raised early in the week on the arrival of the transport Mobile. The newspapers were full of accounts of the horrors of the trip and the statement was reiterated that "salt water was used to wash the soldiers' wounds."

When the leading editorial in this issue was written there had been no reports received on the conduct of the Seneca, and Concho transports in response to demands made by yellow journals—newspapers and medical publications—chief among the latter was the *Medical Record* of New York which one week condemned the whole medical department; the next, devoted space to the heroism of the surgeons and flattered the medical corps and then the week following, like the good cow with bad habits, kicked over its pail of milk by again abusing the corps and its chief, Surgeon-General Sternberg.

Since then, however, there has been issued an official statement by the medical department which, in brief, shows that this department was not responsible for the arrangements for carrying home the troops. This statement is not controverted and places the burden on the war department, especially that division presided over by the Quartermaster-General. This official has issued a personal statement exonerating all officers of his department at this end, and so leaves the matter of responsibility at the door of the Quartermaster officer at Santiago, who was acting directly under Gen. Shafter's orders.

The case of the Mobile, however, seems to have called for immediate action. Complaint was made that the vessel was sent away from Cuba with insufficient supplies and too few medical officers.

President McKinley received a great many letters of complaint, some general, some specific and he immediately ordered an investigation. He placed the matter in the hands of Col. F. B. Hecker, chief of the transportation department, with orders to make a personal report at the earliest possible moment.

---

It is amazing that medical journals in such considerable numbers should follow the lead of the saffron-hued newspapers in their attacks on the management of the army medical department when there is not the slightest justification for such abusive criticism. When it is remembered that we have vanquished a defiant and boastful foe on land and sea, in Orient and Occident, creating and fashioning the tools wherewith to do the work in such a comparatively short time and doing the work with such small aggregate loss, we should rather find occasion for praise than blame of all the staff departments of the army and navy. It is a certain fact that never in the annals of war has so much been accomplished with so little loss in men and material, and in after years when history shall be calmly written it will be found that the medical department of the army, under the leadership of Surgeon-General Sternberg, will be accorded a proud place.

It is not the soldiers that complain, but civilians, unskilled in war and unknown to war's needs. Colonel John Jacob Astor has offered the most lucid explanation of the difficulties surrounding the Santiago campaign in general and of getting medicines and supplies to the sick and wounded in particular. He stands loyally by General Shafter, his chief, and says, according to the *Chicago Record:*

> That it was nobody's fault in particular. There were very few boats attached to the transports for landing, the lighters which were to have been used for that purpose having been lost at sea during the voyage from Tampa. With these few boats the soldiers were taken ashore as rapidly as possible. The next thing in importance was the ammunition and provisions, which were absolutely necessary, and as the men pushed on toward Santiago it required every resource at the command of the quartermasters to supply them with food. For several days they were very short. The roads were impassable for wagons, pack mules were scarce and everybody connected with the quartermaster and commissary departments was working day and night to push forward the ordnance and commissary supplies. They were, therefore, deaf to the clamors of the surgeons, who came ashore without their instruments and medicines, and then had no means of going back for them. As fast as a transport was unloaded it was sent away, in order that another might take its place. In that way, Colonel Astor says, the surgeons and their supplies became separated, but it was not their fault any more than it was the fault of other staff officers. The facilities were very limited, but everybody did the best he could.

---

It is not often that a medical journal receives such courteous recognition from a nonprofessional source as is contained in the following editorial in the *Sunday News* of August 21, 1898. It is written by the editor and owner himself, and we reproduce it entire out of consideration for our associates with an apology for publishing in our own journal such a flattering personal for ourselves :

A PROGRESSIVE MEDICAL JOURNAL.

The BUFFALO MEDICAL JOURNAL is making rapid strides to the front. An effort will now be made to extend its usefulness by giving it a wider and more extensive circulation. So far as mint goes and range of subjects discussed, it equals any medical journal published in this country. The editors are Dr. William Warren Potter and Dr. Thomas Lothrop, both men of high professional standing and great prominence in the world of medicine. Dr. Potter is one of the best-known physicians in the United States. He is president of the state board of medical examiners and an officer in many American associations. To his efforts is largely due the state control of physicians. He has fought misrepresentation and chicanery for years and the present high standard of medical education is largely due to his efforts as an editor and a physician.

Dr. Thomas Lothrop was one of the moving spirits in the University of Niagara's medical department and he established the Woman's hospital at Georgia and Seventh streets. There is a board of associate editors concerned in the conduct of the MEDICAL JOURNAL, composed of Dr. Parmenter, professor of anatomy; Dr. James W. Putnam, professor of nervous diseases, and Dr. Ernest Wende, health commissioner of Buffalo and professor of dermatology in the University of Buffalo, and Dr. A. A. Hubbell, Dr. William C. Krauss and Prof. John Miller, who held professorships in the University of Niagara. By this it will be seen that the BUFFALO MEDICAL JOURNAL is written by men of high professional attainments. A new name appeared in the August number of the JOURNAL, when it was announced that the surgical department would be conducted by Dr. John Parmenter, assisted by Dr. Nelson W. Wilson.

Dr. Wilson, who was for several years before his graduation from the University of Buffalo connected with the editorial staff of the *News*, is a sharp, terse writer and knows how to make the most unattractive theme interesting to the general reader by the skilful manner in which he handles it.

---

EVERY Buffalonian is proud of his city and wants to see it become more and more metropolitan in as many directions as possible compatible with good morals, good appearances and good health. There is a limit, however, to the metropolitan aspect of torn-up streets and the Board of Public Works should not overlook the fact that a wholesale ripping up of the streets of a city is not the best thing in the world for good health. The gas companies are the most frequent disturbers of the streets.

The JOURNAL stands for good health for Buffalo and believes that the Board of Public Works should regulate the digging up of streets by corporations. We want to see Buffalo metropolitan, but we want it healthy first of all.

---

THE death of two men while riding their wheels in one week, as happened in Buffalo during the third week of August, ought to prove a warning to even enthusiastic cyclists. It is true in both the cases mentioned the victims were advanced in years—77 and 61 respectively—and no doubt organic heart changes were taking place, but there was all the more reason why they should have abandoned their wheels on hot days. But it is doubtful if anything in the way of warning, no matter how forceful, will prevail against the excited and unreasoning bicycle devotee.

